# Editorial: Therapeutic and protective approaches to gastrointestinal tract infections

**DOI:** 10.3389/fphar.2025.1650144

**Published:** 2025-07-30

**Authors:** Roland Bücker

**Affiliations:** Clinical Physiology and Nutritional Medicine, Clinics of Gastroenterology, Infectious Diseases and Rheumatology, Charité—Universitätsmedizin Berlin, Berlin, Germany

**Keywords:** antibiofilm compounds, drug repurposing, antimicrobials, antibiotics, natural compounds

The burden of infectious diseases on the digestive and excretory systems remains high. The integrity of the gastrointestinal tract and its barrier mechanisms against pathogens (such as the protective microbiome, the mucous layers, the epithelial barrier, and the mucosal immune system) supports the defence against infections. However, from time to time, medical intervention using pharmacological approaches is necessary.

This Research Topic aims to explore new and existing treatment methods (off-label) that can reduce symptoms by improving the mucosal homeostasis, the microbiota, the epithelial barrier function, and/or the immune system. Another focus is on the treatment of gastroenteritis and pharmacological strategies against biofilm formation and their antimicrobial efficacy. Human pathogens can originate from animals and cause acute inflammation in the intestine (*Campylobacter jejuni* (Sharafutdinov et al., 2025)). Related ε-proteobacteria, for which humans are the only source of infection, have the unique ability to chronically colonize the stomach (*Helicobacter pylori* (Krzyżek et al., 2024)). In addition, human-transmitted chronic infection of the liver (HBV (Huang et al.)) or acute infection of the gut (*Clostridioides difficile* (Wang et al., 2025)) are prominent examples of infections of the digestive system. Moreover, some pathogens invade from the environment and can reside in the gastrointestinal tract (*Escherichia coli, Staphylococcus aureus, Klebsiella* spp., and *Enterobacter* spp. (Quan et al., 2024; Truong and Mudgil, 2023; Zaimović et al., 2022)). From there, they can spread to other organs (such as *E. coli* in urinary tract infections) if the intestinal barrier is weak and/or the immune system is unable to kill all pathogens from the bloodstream. Regardless of the route of infection or which organ is affected, treatment with pharmaceuticals is usually unavoidable. To improve understanding and treatment options for the protection of the digestive and excretory system, we have summarized contributions to this Research Topic.

*Campylobacter jejuni* is considered the most common cause of bacterial gastroenteritis worldwide and is also one of the most common causes of bacterial zoonoses of the gastrointestinal tract. The usual treatment is only a symptomatic treatment, for example, through oral rehydration. The administration of antibiotics is only recommended in the case of severe symptoms of infection or in patients who are prone to infections. In recent years, research has been conducted to identify alternative prevention and treatment strategies, as summarized in the included review (Sharafutdinov et al., 2025). Several potential natural therapeutic compounds with pharmacological efficacy are listed here, such as polyphenols like resveratrol, prohormones like vitamin D, or antimicrobials with direct inhibitory effects on bacteria. A number of pharmaceutical compounds were shown to reduce *C. jejuni*-mediated epithelial cell damage, inflammation, and epithelial cell apoptosis induction (Sharafutdinov et al., 2025). The phylogenetic group of *Campylobacteriaceae* is large and includes over 30 described species, most of which are suspected of being pathogenic to humans.

The best-known pathogenic relative of this group is *H*. *pylori*, the causative agent of gastritis, gastric and duodenal ulcers, and stomach cancer, which fortunately can be prevented by eradication therapy combining proton pump inhibitors and antibiotics during the infection. In antibiotics research, the modulatory effect of sub-minimal inhibitory concentration values of antibiotics on the biofilm development of *H. pylori* has been described (Krzyżek et al., 2024). The biofilm-modulating impact of sub-minimal inhibitory concentrations of metronidazole and levofloxacin consisted of an induction of adaptive changes in the fatty acid profile of the bacterial membrane, as well as stimulation of auto-aggregation and modulation of the amount of extracellular matrix proteins. When *H. pylori* was exposed to sub-minimal inhibitory concentrations of clarithromycin, the opposite effect was observed. Thus, clarithromycin constitutes an antibiotic with a promising biofilm-preventing activity against *H. pylori*. Moreover, the current data highlights the importance of maintaining appropriate concentrations of antibiotics during treatment of *H. pylori* as a crucial factor for the therapeutic effect (Krzyżek et al., 2024). Without eradication, after years of chronic colonization with *H. pylori*, gastric cancer can develop as a serious consequence of the infection.

Another infection that can lead to cancer is chronic hepatitis B. The hepatitis B virus (HBV) infection is clearly a risk factor for the development of liver cancer (hepatocellular carcinoma). For combating this virus infection antiviral therapies including interferons and nucleoside/nucleotide analogues are performed. In a clinical study, the importance of maintaining a 48-week overall virus response (VR) target in patients without high virus load (HVL) was shown (Huang et al.). For patients with both HVL and hepatitis B e-antigen-positive status, extending the antiviral monotherapy with entecavir or tenofovir duration to 96 weeks was recommended. This recommendation was supported by the relatively lower VR rates at 48 weeks, the lack of significant differences in disease progression and maintained virologic response achievement rates, as well as their generally favourable prognosis, which is characterized by a low incidence of disease progression (Huang et al.). Thus, the suggestion to extend the antiviral treatment to 96 weeks was the relevant information of the study to change the clinical practice.

Although statins are primarily approved for cardiovascular use, they have also been shown to affect outcomes in acute renal failure, venous thromboembolism, inflammatory bowel disease, autoimmune diseases, and malignancies such as hepatocellular carcinoma.

Besides the positive effects of statin therapy in hepatocellular carcinoma, statins can influence infections such as *Clostridioides difficile*-induced enteritis (CDE) since, beyond their traditional lipid-lowering effects, they also possess anti-inflammatory and immunomodulatory properties. Statins are pharmaceuticals widely used as lipid-lowering drugs against atherosclerosis, among them rosuvastatin or simvastatin, which are inhibitors of the 3-hydroxy-9-methylglutaryl-coenzyme A reductase. In a clinical study, the positive side effects of statins on CDE were investigated (Wang et al., 2025). It was shown that statin administration can reduce the risk for patients with CDE to be admitted to the intensive care unit (ICU), but statins did not decrease the in-hospital mortality rate for such patients (Wang et al., 2025). This study provides a more reliable basis for the additional administration of statins for the prevention and treatment of CDE. As a mechanism for reduced CDE incidence in statin users, the authors hypothesize that the effect is partly attributed to the immunomodulatory action of statins, which enhances the function of neutrophils and phagocytes and their capacity to produce extracellular traps. For the second finding—unchanged mortality—the authors assume that patients with CDE in the ICU frequently present with additional severe comorbidities, which may directly contribute to death rates (Wang et al., 2025). The treatment standard of CDE is antibiotic therapy with vancomycin or fidaxomicin. In most bacterial infections of the digestive or excretory system, the use of antibiotics is the gold standard.

In a systematic review with meta-analysis, the efficacy of novel β-lactam antibiotics and other antibiotics such as carbapenems against urinary tract infection (UTI) was compared (Quan et al., 2024). Novel β-lactam antibiotics were found to have similar clinical cure and adverse effects to other antibiotics in the treatment of complicated UTIs. In a subgroup analysis, novel β-lactam antibiotics showed higher clinical cure rates at the end of the treatment compared to carbapenems and showed better microbiological response than other antibiotics (Quan et al., 2024). The safety of novel β-lactam antibiotics was similar to that of other antibiotics (Quan et al., 2024). The population studied consisted predominantly of individuals with acute pyelonephritis. *Escherichia coli* emerged as the predominant pathogen in the studies examined, leading to enhanced clinical and microbiological response rates (Quan et al., 2024). Uropathogenic *E. coli* mostly originates from the human gut and is translocated from the intestine into the excretory system via the bloodstream or as an ascending infection from the urethra. Carbapenemase-producing *E. coli* infections have become a global public health threat and are associated with high morbidity and mortality in internal medicine. These results support the use of new β-lactam antibiotics as a potential alternative to carbapenems in patients with carbapenem-resistant Gram-negative infections. The identification of alternative effective antibiotics is therefore crucial.

Compared to antibiotic administration, the direct efficacy of natural compounds, such as lavender essential oil (LEO), against bacteria was demonstrated in another systematic review (Truong and Mudgil, 2023). The antibacterial efficacy of LEO, a natural remedy from traditional medicine, was elucidated. LEO appears to have an antimicrobial effect on some strains of *S. aureus* and methicillin-resistant *S. aureus* (MRSA). Although several studies have observed an antimicrobial effect on *S. aureus* when LEO was used alone, the ranges and conditions of antimicrobial efficacy vary. Some studies showed negligible efficacy, while others showed significant efficacy (Truong and Mudgil, 2023). LEO also appears to act synergistically with other antimicrobial agents, such as hydroxyapatite, octenidine, other essential oils, or other antibiotics (Truong and Mudgil, 2023). Therefore, it might be interesting to further investigate compounds that show synergistic effects with LEO or to test other potential active ingredients for synergistic activity.

Further antibacterial compounds, such as pyrazole derivatives and their copper II complexes, were tested for antimicrobial effects (Zaimović et al., 2022). Pyrazole derivatives such as 4-bromo-2-(1H-pyrazol-3-yl)phenol, 4-nitro-3-pyrazolecarboxylic acid, or N-(benzyloxycarbonyl)-1H-pyrazole-1-carboxamidine, and selected complexes of Cu(II) with the mentioned pyrazoles as ligands, were used as bioactives for testing of antibacterial activity. Some pyrazole compounds have shown inhibitory effects against the growth of *E. coli*. A small number of compounds showed inhibitory effects against the growth of *Klebsiella* spp. and *Enterobacter* spp., but none of the pyrazoles showed any inhibitory effect on *S. aureus* compared to amoxicillin as a standard medication (Zaimović et al., 2022). The results also show how changing the concentration of the same compound affects the inhibition, particularly by reducing the concentration of pyrazole. This compound changes from a fairly active to an inactive compound, observed in the *Klebsiella*–*Enterobacter* test group (Zaimović et al., 2022). These derivatives could be promising molecules for the further development of antimicrobial agents.

The investigated compounds described in this Research Topic were tested for proof of principle against a specific pathogen or for the applicability of substances on a larger scale. Drug development in medical research was described for novel β-lactam antibiotics or pyrazole derivatives, for example. Drug repurposing was suggested for off-label use of statins in CDE. Moreover, the interference of compounds with microorganism adherence and biofilm formation adds up as a therapeutic approach. Furthermore, modulation of the immune response and anti-inflammatory activity of compounds is another preventive and therapeutic mechanism in treatment of infectious diseases. For pharmacological research in infectious diseases, it is advantageous to combine different scientific disciplines in order to improve the translation of therapeutic approaches from the laboratory to practice.

This Research Topic summarises new research findings and reviews on various pharmaceutical approaches to infections of the digestive or excretory system. The papers contribute to our understanding of various aspects of the complex nature of infectious diseases and their treatment, and they call for further research on the mode of action of essential substances in internal medicine.
